# Pharmacological inhibition of BCL-2 with the FDA-approved drug venetoclax impairs longitudinal bone growth

**DOI:** 10.1038/s41598-023-34965-4

**Published:** 2023-05-17

**Authors:** Lilly Velentza, Malin Wickström, Per Kogner, Claes Ohlsson, Farasat Zaman, Lars Sävendahl

**Affiliations:** 1 Division of Pediatric Endocrinology, Department of Women’s and Children’s Health, Karolinska Institutet, Visionsgatan 4, BioClinicum J9:30, SE-171 64 Stockholm, Sweden; 2 Childhood Cancer Research Unit, Department of Women’s and Children’s Health, Karolinska Institutet, Stockholm, Sweden; 3 Astrid Lindgren Children’s Hospital, Karolinska University Hospital, Stockholm, Sweden; 4 Centre for Bone and Arthritis Research, Department of Internal Medicine and Clinical Nutrition, Institute of Medicine, Sahlgrenska Academy, University of Gothenburg, Gothenburg, Sweden

**Keywords:** Cell biology, Endocrinology, Oncology

## Abstract

Treatment-related skeletal complications are common in childhood cancer patients and survivors. Venetoclax is a BCL-2 inhibitor that has shown efficacy in hematological malignancies in adults and is being investigated in pediatric cancer clinical trials as a promising therapeutic modality. Venetoclax triggers cell death in cancer cells, but whether it exerts similar effects in normal bone cells, is unknown. Chondrogenic ATDC5 cells, E20 fetal rat metatarsal bones, and human growth plate biopsies were treated with different concentrations of venetoclax. Female NMRI nu/nu mice were treated with venetoclax or vehicle for 15 days. Mice were X-rayed at baseline and at the end of the experiment to assess longitudinal bone growth and body weight was monitored throughout the study. Histomorphometric and immunohistochemical analyses were performed to evaluate treatment effects on the growth plate cartilage. Venetoclax decreased the viability of chondrocytes and impaired the growth of ex vivo cultured metatarsals while reducing the height of the resting/proliferative zone and the hypertrophic cell size. When tested in vivo, venetoclax suppressed bone growth and reduced growth plate height. Our experimental data suggest that venetoclax directly targets growth plate chondrocytes suppressing bone growth and we, therefore, encourage careful monitoring of longitudinal bone growth if treating growing children with venetoclax.

## Introduction

The survival rates for childhood cancer have been significantly improved over the past few years, mainly due to effective chemotherapy and radiotherapy. Yet, managing treatment-related adverse effects remains a challenge^1^. Among the different organ systems, the growing skeleton can be severely affected during and after cancer treatment, leading to growth impairment, bone mass deficits, fractures and chronic pain^2–4^. It has been recently shown in a large multicenter retrospective study that skeletal-related adverse events were more common in childhood cancer survivors who also faced an increased risk for hospitalizations due to skeletal morbidities up to the age of 60 years^5^.

Although bone health impairment in the childhood cancer setting is multifactorial, previous studies have reported direct deleterious effects of anticancer regimens on the growth plate, which is the main site of longitudinal bone growth^6–9^. The growth plates are cartilaginous structures located near the ends of long bones and consist of three zones of chondrocytes: the resting, proliferative and hypertrophic zone which will eventually be replaced by bone cells during the process called endochondral ossification^10,11^. During this process, the chondrocytes in the growth plate undergo progressive proliferation and differentiation, each regulated by numerous molecules and signaling pathways^12^. Any factor- including chemotherapeutic drugs- that negatively interferes with this tight organization and regulation of the growth plate cartilage, may result in bone growth suppression in developing children.

As novel compounds for the treatment of childhood cancer are continuously explored and launched into clinical practice, the investigation of their side effects is of utmost importance for childhood cancer patients, who are vulnerable to treatment-related toxicities. One of the upcoming therapeutic strategies that has gained much attention during the past years is targeting the BCL-2 protein with selective antagonists. Venetoclax is an orally administered FDA-approved BCL-2 inhibitor that has shown antitumor efficacy in adult patients^13^ as well as in preclinical models of pediatric malignancies^14–17^. Venetoclax is currently under investigation in children and young adults diagnosed with relapsed/refractory solid or hematological cancers and is administered either as monotherapy or in combination with conventional chemotherapy^18,19^. The mechanism of action involves the selective inhibition of BCL-2 that is aberrantly expressed in the cancer cells, resulting in cell death by apoptosis^20^. However, BCL-2 is also crucial for the survival of various cell types, including bone cells and plays an important role during normal development and bone growth^21^. Previous studies have shown that ablation of the Bcl2 gene in mice resulted in severe organ defects and impaired bone growth^22–24^. Taken together, it is not yet clear if pharmacological inhibition of BCL-2 with venetoclax could exert deleterious side effects on normal cells in the bone tissue and any negative effects on the growth plate cartilage remain to be investigated.

The primary objective of the present study was to explore the effects of venetoclax on longitudinal bone growth and for this purpose, we applied in vitro, ex vivo and in vivo experimental models of childhood growth.

## Methods

### Cell culture

The mouse chondrogenic ATDC5 cell line (RIKEN Cell Bank, Ibaraki, Japan) was maintained in DMEM/F12 medium (Gibco) containing 5% FBS and subcultured every 2–3 days. The cells were tested for mycoplasma with the LookOut® Mycoplasma PCR Detection Kit (Sigma-Aldrich).

### Cell viability assay

ATDC5 cells were plated at 2000 cells per well in 96-well plates. After reaching 80% confluency, the medium was changed to test medium (1% FBS), containing Venetoclax (Ven) at the concentrations of interest or dimethyl sulfoxide (DMSO) for the control group. Cell viability after 48-h or 72-h treatment was assessed with the CellTiter-Glo® Luminescent Cell Viability Assay (G7570; Promega) according to the manufacturer’s protocol. Luminescent signal was measured with a FLUOstar® Omega microplate reader (BMG LABTECH). For each treatment well, the blank was subtracted, and cell viability was expressed as percent relative to untreated control. Three independent experiments were performed, and each concentration was tested in triplicates.

### Ex vivo culture of fetal rat metatarsals

Sprague–Dawley pregnant rats were purchased from Janvier labs at E13 and were housed in the animal facility for a 7-day adaptation period before euthanasia via carbon dioxide (CO_2_) to obtain the fetuses and collect the hind paws. The three middle metatarsals were microdissected from the hind paws and cultured in 24-well plates as previously described^25^. The culture medium used was DMEM/F12 (Gibco) supplemented with 50 μg/ml ascorbic acid (Sigma-Aldrich), 1 mM sodium glycerophosphate (Sigma-Aldrich), 0.2% bovine serum albumin (BSA; Sigma-Aldrich) and 20 μg/ml gentamicin, including venetoclax (1 nM-10 μΜ) or DMSO for the control group. Culture medium changes together with an image capture of the bones (Hamamatsu C4742–95 digital camera mounted on a Nikon SMZ-U microscope) were performed on days 0, 2, 5, 7, 9 and 12 (termination of culture) and the bone length was measured with the Infinity Analyze software (Lumenera Corporation). Bone growth was expressed as % length increase from day 0 (start of the culture). Three independent experiments were performed, and each metatarsal was considered an independent observation. After termination of the experiment, the metatarsals from each group were washed in PBS, fixed in 4% paraformaldehyde (PFA) for 24 h, and embedded in paraffin for further analyses.

### Ex vivo culture of human growth plate tissues

Growth plate biopsies were collected from the proximal tibia and distal femur of children undergoing epiphyseal surgery due to constitutional tall stature, as previously described^26^. The main patient characteristics are summarized in Supplementary Table 2(S2). Right after collection, the biopsies were put in DMEM-high glucose medium without phenol red (Sigma-Aldrich) supplemented with 10 µg/ml gentamicin and kept on ice. Thereafter, the tissues were cut into thin slices (0.5–1 mm) and each piece was individually transferred into 24-well plates containing culture medium (DMEM-high glucose without phenol red supplemented with 10 µg/ml gentamicin, 50 µg/ml ascorbic acid, 1 mM beta-glycerophosphate, and 0.2% BSA) where the compound of interest was added. For each treatment group, up to 4 pieces were used and the experiment was terminated after 48 h of culture. After that, the biopsies were washed with PBS, fixed in 4% PFA for 24 h, decalcified in EDTA, and put in 70% ethanol until paraffin embedding and sectioning. Each biopsy piece was considered an individual observation.

### In vivo studies

6-week-old female NMRI nude (BomTac: NMRI-*Foxn1*^*nu*^) mice were supplied by Taconic Biosciences and left to acclimatize for a 7-day adaptation period in the animal facility. Water and standard food pellets were provided ad libitum. To ensure that all groups had similar mean weights at baseline, mice were stratified into the different treatments based on their body weight and were treated with venetoclax (100 mg/kg/day via oral gavage, *n* = *5* or 200 mg/kg/day via oral gavage, *n* = *5*) or vehicle solution (60% PHOSAL 50PG, 30% PEG-400, 10% ethanol, *n* = *4*) for 15 consecutive days. Body weight was measured 2–3 times per week and general health status was monitored daily for any signs of treatment-related discomfort. To monitor bone growth, which was the primary endpoint of the study, X-ray imaging (GE AMX-4, GE Healthcare, USA, with the settings: 50 kV, 2.5 mAs) was performed at baseline and endpoint, after mild isoflurane anesthesia (2.5–4%), as previously reported^7^. The length of the right tibia bones was measured in a blinded manner. The number of mice per group was decided based on previous experience from our group.

### Alcian blue staining

For histomorphometry analysis, paraffin sections from fixed metatarsal or tibia tissues (5 μm) were deparaffinized in xylene, rehydrated in graded ethanols, and stained with Alcian blue solution (1% in 3% acetic acid, pH 2.5, Sigma-Aldrich) for 10 min, followed by counterstaining with Nuclear Fast Red solution (Sigma-Aldrich) for 10 min.

### Quantitative histology of rat metatarsals and mouse tibial growth plates

The resting and proliferative zone (R + P) height was measured at ten different regions of the metatarsal growth plate, both at the proximal and distal ends. A cell was considered hypertrophic if it was larger than 7 μm and the cell size was calculated based on the mean size of 20 hypertrophic cells per metatarsal bone (both at the proximal and distal end). The height of the mouse tibial growth plate and different zones were measured in at least ten regions, and the mean value for each bone was calculated. All analyses were performed in a blinded manner using the Image J Software^27^.

### Immunohistochemistry

Paraffin-embedded bone sections were deparaffinized and rehydrated, followed by heat-induced antigen retrieval with citrate buffer (10 mM, ph 6.0). Endogenous peroxidase was blocked with 3% H_2_O_2_ in methanol and blocking was performed with goat serum in 2% BSA for 1 h. The sections were incubated with the primary antibody of interest at 4 °C overnight. For negative controls, one slide was incubated with unspecific IgG of the same species as the primary antibody. Next, after washing with PBS-T for 20 min, the secondary antibody was applied for 1 h, followed by incubation with horseradish peroxidase (HRP)-conjugated streptavidin (VECTASTAIN® ABC-HRP Kit, VECTOR Laboratories) for signal detection. 3′-Diaminobenzidine (DAB, Sigma-Aldrich) was used as chromogenic substrate and Alcian blue solution (1% in 3% acetic acid, pH 2.5, Sigma-Aldrich) as counterstain. To ensure that all samples were handled under the same conditions, bones from all treatment groups were mounted on the same slide. Photos of each slide were taken with a microscope (Leica Microsystems) and quantification of protein expression was performed with Image J Software, using the IHC Toolbox plugin^28^. For the human growth plate tissue, quantification was done by measuring the number of positive cells per mm^2^ of growth plate area. The antibodies used are listed in Supplementary Table 1(S1).

### Statistical analysis

All statistical analyses were performed in GraphPad Prism 9.1.1. A 2-tailed unpaired *t* test was applied when 2 groups were compared, whereas one-way ANOVA followed by Holm-Sidak post-hoc testing was used when comparing multiple groups. 2-way ANOVA was used for the analysis of the effect of venetoclax treatment over time in the fetal metatarsal experiments. For all analyses, a p-value < 0.05 was considered statistically significant.

### Ethics declarations

All animal studies were approved by the local ethics committee (Stockholm North Animal Ethics Committee, Permits No. 13820-2019 and No. 13572-2018) appointed under the control of the Swedish Board of Agriculture and the Swedish Court and were conducted in full compliance with the Directive 2010/63/EU, the national regulations and ARRIVE guidelines (https://arriveguidelines.org). The local ethics committee approved the collection of human growth plate biopsies (Karolinska Institutet Research Ethics Committee North at the Karolinska University Hospital, Stockholm, Sweden, Permit No. 97-214). Informed consent was obtained from each patient and their legal guardians and documented in the original hospital records. We confirm that all experiments were carried out in accordance with relevant guidelines and regulations.

## Results

### Venetoclax treatment reduced the viability of cultured ATDC5 cells

**Figure 1:**
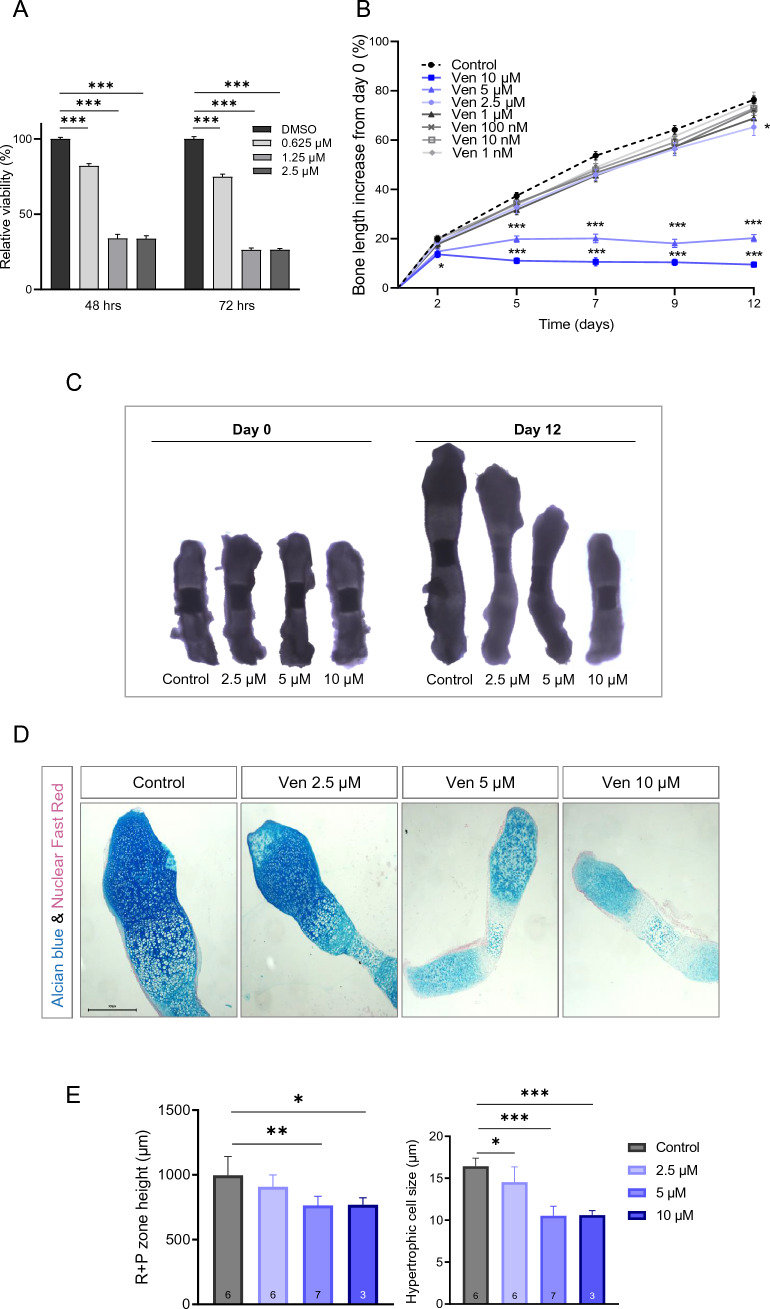
Effects of Venetoclax (Ven) on ATDC5 cells and E20 metatarsal bones. (**A**) Venetoclax decreased the viability of chondrogenic ATDC5 cells after 48 or 72 h of treatment. Three independent experiments were performed. Mean values ± SEM are presented. ***p < 0.001. (**B**) Treatment with venetoclax impaired bone growth ex vivo. E20 fetal rat metatarsal bones were treated with increasing concentrations of venetoclax and their growth was monitored for 12 days in culture. The graph shows the increase (%) in bone length over time from day 0. Venetoclax treatment caused dose-dependent growth retardation compared to the control group. Mean values ± SEM from three independent experiments are presented. *p < 0.05, ***p < 0.001, n = 15/group. (**C**) Microscopic images of fetal rat metatarsals cultured ex vivo with venetoclax or DMSO (control) as captured in real-time conditions on day 0 (start of the experiment) and day 12 (termination). (**D**) Representative images of metatarsal sections stained with Alcian blue and Nuclear Fast Red. Venetoclax treatment affected the organization of chondrocytes in the different zones. Magnification 5x, scale bar = 500 μm. (**E**) Quantification of the resting and proliferative (R + P) zone height, and hypertrophic cell size. Venetoclax treatment significantly reduced R + P zone height and hypertrophic cell size. Mean values ± SD are presented. *p < 0.05, **p < 0.01, ***p < 0.001, n = 3–7 bones/group.

To investigate any dose-dependent effects of venetoclax on chondrogenesis, mouse-derived chondrogenic ATDC5 cells were treated with venetoclax (0.625–2.5 μM) for 48 or 72 h. Venetoclax treatment significantly reduced the viability of ATDC5 cells as compared to DMSO-treated cells (control) (Fig. 1A).

### Venetoclax induced growth retardation and morphological changes in ex vivo cultured bones

**Figure 2:**
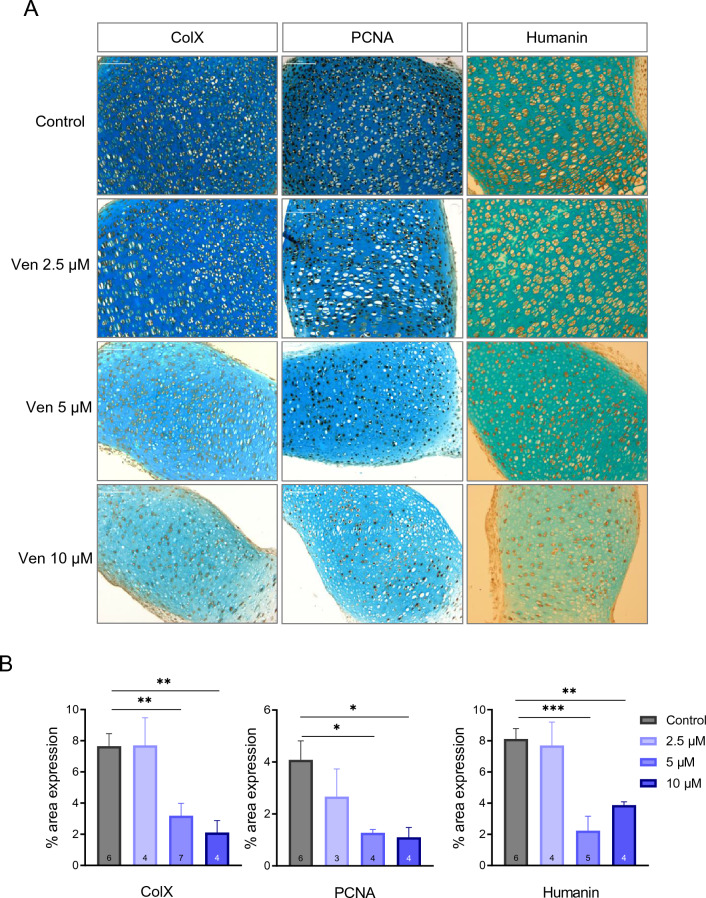
Venetoclax impairs protein expression in fetal rat metatarsals. (**A**) Representative images of metatarsal sections stained for ColX, PCNA, and humanin. Magnification 20x, Scale bar: 100 μm. (**B**) Quantification of PCNA, ColX, and humanin protein expression in E20 metatarsal bones. Mean values ± SEM are presented. *p < 0.05, **p < 0.01, ***p < 0.001, n = 3–7 bones/group.

Next, we used a well-established model of ex vivo cultured fetal rat metatarsal bones to investigate any direct effects of venetoclax treatment on chondrocytes. Venetoclax induced a concentration-depended suppression of metatarsal bone growth compared to the control group, an effect that was already present on day 2 for the 5 μΜ and 10 μΜ groups (Fig. 1B). On day 12 (termination of the experiment), the growth of metatarsals treated with 2.5 μΜ, 5 μΜ, and 10 μΜ was significantly suppressed compared to the control group (Fig. 1B). Furthermore, macroscopic evaluation of the treated metatarsals showed that venetoclax treatment reduced the intensity of Alcian blue staining and caused profound changes in the morphology of the metatarsal growth plates (Fig. 1C,D). More specifically, the length of the resting + proliferative (R + P) zone was significantly reduced in the 5 μM and 10 μM venetoclax groups compared to the control group and the hypertrophic cell size was significantly reduced in all venetoclax-treatment groups (Fig. 1E). Moreover, venetoclax treatment significantly decreased the expression of the hypertrophy marker Collagen X (ColX) and the proliferating cell nuclear antigen (PCNA), as well as the expression of humanin, an endogenously produced mitochondrial peptide which is known to exert cytoprotective effects (Fig. 2). Alterations in the expression of the pro-apoptotic protein Bax were also noted, although the results of the quantification did not reach statistical significance (p = 0.1; Suppl. Fig. 1).

### Venetoclax suppressed longitudinal bone growth in young mice

**Figure 3:**
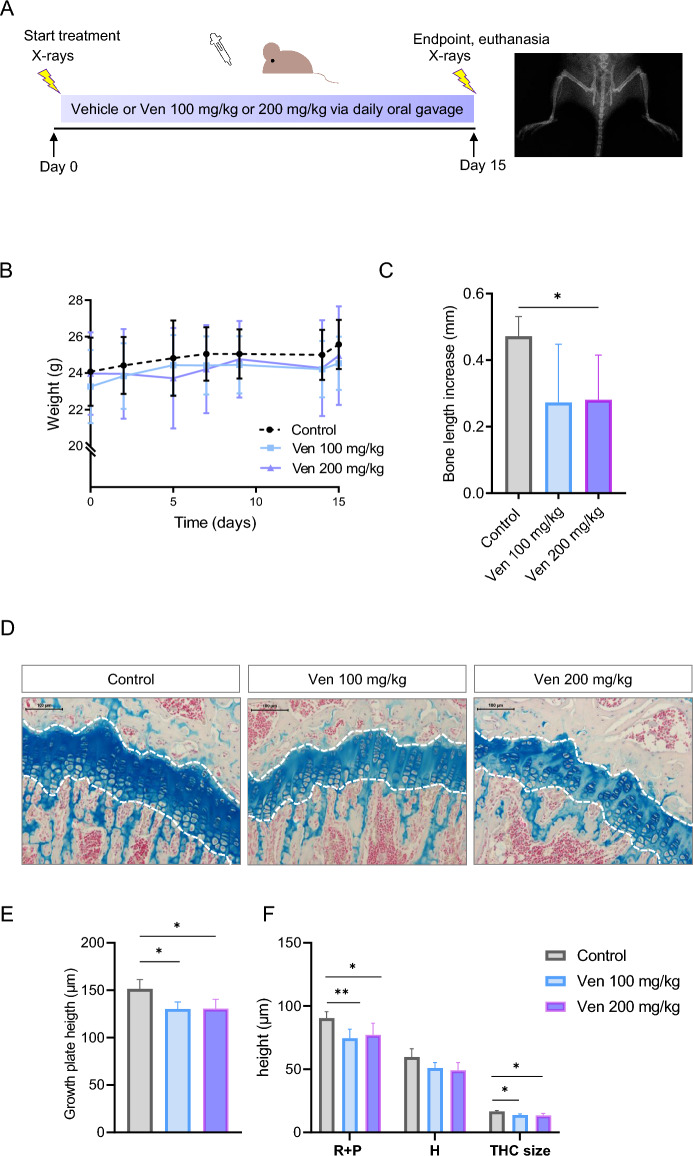
Venetoclax (Ven) caused growth retardation in vivo and reduced growth plate height. (**A**) Schematic illustration of the experimental set-up. 7-week-old female NMRI nu/nu mice were treated with vehicle (control, n = 4), Ven 100 mg/kg (n = 5), or Ven 200 mg/kg (n = 5) for 15 days. X-rays were performed at baseline (day 0) and at the endpoint (day 15). (**B**) Weight gain of the treated mice over time. No difference among the different treatment groups was detected. Mean values ± SD are presented. (**C**) Venetoclax treatment impaired bone growth in vivo. Mean values ± SD are presented. *p < 0.05, n = 4–5/ group (**D**) Representative images of proximal tibia growth plates stained with Alcian blue/Nuclear Fast Red solution for analysis of growth plate histomorphometry. Growth plate height was reduced in venetoclax-treated mice. Magnification 20x, scale bar = 100 μm. (**E**,**F**) Quantification of the histomorphometric analyses of the mice growth plates. The total growth plate height (μm), the height of R + P zone and the size of the terminal hypertrophic cell (THC) were reduced in both venetoclax-treated groups, compared to the control group. Mean values ± SD are presented. *p < 0.05, **p < 0.01, n = 4–5/group. *H* Hypertrophic.

Based on our in vitro and ex vivo findings, we then aimed to investigate the effects of venetoclax treatment in vivo. Seven-week-old female NMRI nu/nu mice were treated with either venetoclax 100 mg/kg (*n* = *5*), venetoclax 200 mg/kg (*n* = *5*), or vehicle (*n* = *4*) by daily oral gavage for 15 days and X-rayed at the start (day 0) and end (day 15) of the treatment to monitor bone growth (Fig. 3A). Body weight did not differ among the treatment groups (25.5 ± 1.3 g in control; 24.5 ± 1.4 g in 100 mg/kg; 24.9 ± 2.7 g in 200 mg/kg; Fig. 3B). Bone growth was decreased in animals treated with venetoclax 100 mg/kg or 200 mg/kg (Fig. 3C; p = 0.069 and p < 0.05, respectively vs. control). Morphometric analysis of the growth plates showed that mice treated with both doses of venetoclax had significantly reduced growth plate height compared to the control group, with a more pronounced effect on the R + P zone (Fig. 3D–F). Changes in the expression of PCNA, Bax, caspase-3 and ColX did not reach statistical significance (Suppl. Figs. 2–4).

### Effects of venetoclax on the human growth plate cartilage

**Figure 4:**
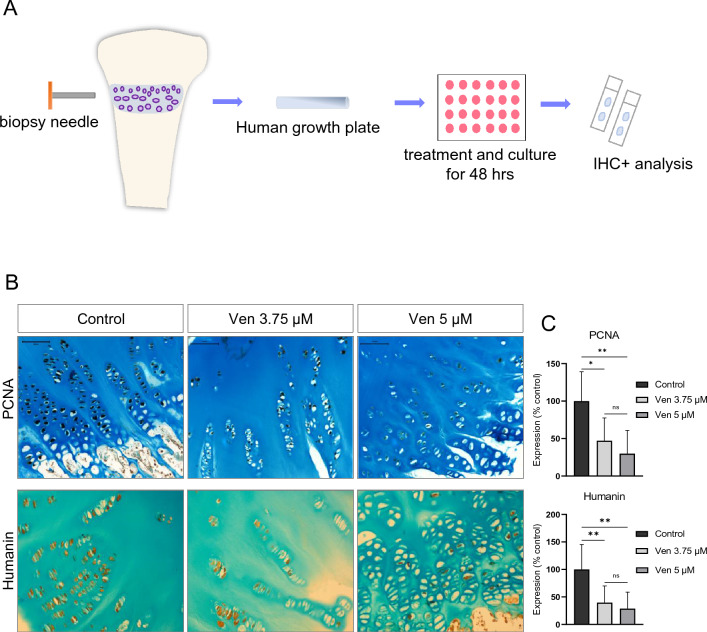
Effects of venetoclax on the human growth plate. (**A**) Illustration showing the procedure of human growth plate tissue collection. A biopsy needle is used during the epiphysiodesis surgery to collect growth plate tissue from the tibia or femur. The tissue is then cut into small pieces which were individually placed in 24-well plates in medium supplemented with the drug of interest and cultured ex vivo. After 48 h the culture is terminated, the growth plate pieces are fixed in 4% PFA, decalcified in EDTA, and embedded in paraffin blocks for sectioning. IHC stainings are performed to analyze the expression of different markers. *IHC* immunohistochemistry. (**B**) Representative images of human growth plate tissue biopsies treated with venetoclax (Ven) or DMSO (control) for 48 h. Alcian blue counterstain. Magnification 20x, scale bar = 100 μm. (**C**) Quantification of PCNA and humanin expression in the ex vivo treated human growth plate biopsies. Results of 3 patients are presented. Mean values ± SD are shown. *p < 0.05, **p < 0.01.

Human growth plate biopsies obtained from three patients were treated with venetoclax or DMSO (control group) and cultured ex vivo for 48 h (Fig. 4A). Individual patient characteristics are presented in Supplementary Table 2. Immunohistochemical staining showed that venetoclax in both concentrations impaired the expression of PCNA and endogenous humanin compared to the control group (Fig. 4B,C).

## Discussion

Advances in the development of targeted anticancer strategies have significantly altered the therapeutic landscape of childhood cancer over the past years. Since novel therapies are continuously introduced in clinical practice, the identification of potential toxicities and side effects is of utmost importance for childhood cancer patients. In the present preclinical study, we showed that venetoclax, a selective BCL-2 inhibitor, targeted the growth plate chondrocytes and suppressed longitudinal bone growth.

By applying a well-established model of ex vivo culture, we were able to study the direct effects of venetoclax on fetal rat metatarsal bones under real-time conditions and in the absence of any systemic factors. Interestingly, venetoclax caused dose-dependent growth retardation and disruption of the histological bone phenotype including reduced height of the R + P zone and reduced size of hypertrophic chondrocytes. Growth retardation and disorganization of growth plate chondrocytes have been previously reported in studies where metatarsals were treated ex vivo with other drugs, including proteasome inhibitors and glucocorticoids^6,7,29^. Interestingly, our findings suggest that venetoclax impairs differentiation and proliferation of growth plate chondrocytes, effects that mechanistically have been related to the induction of bone growth retardation^30^. Our ex vivo studies were also expanded to the use of rare human growth plate biopsy material, where venetoclax was confirmed to reduce chondrocyte proliferation and, interestingly also downregulate the chondrocyte expression of humanin. Humanin is an anti-inflammatory mitochondrial peptide that has been associated with longevity exerting anti-inflammatory, anti-oxidative, and anti-apoptotic effects in various tissues, including the brain and heart^31,32^. Notably, disruptions of humanin signaling have been identified as an underlying mechanism in dexamethasone-induced bone growth retardation in cultured rat metatarsal bones and in vivo in young mice^7^.

When performing in vivo studies in female mice, we confirmed a growth-suppressive effect of venetoclax treatment. Furthermore, we found that the growth plate height was reduced in animals treated with venetoclax similar to what has been previously reported after treatment with various cytotoxic drugs, including dexamethasone, bortezomib and doxorubicin^6,7,33^. Similar to our findings in fetal rat metatarsals, both the R + P zone height and the size of terminal hypertrophic cells were significantly reduced in the growth plates of venetoclax-treated mice, indicating that pharmacological BCL-2 inhibition with venetoclax disrupts chondrocyte hypertrophy and proliferation.

The observed adverse effects of venetoclax on bone growth seem to be consistent with previous studies investigating the ablation of the Bcl2 gene in murine models. Interestingly, Bcl2^–/–^ mice demonstrate a pathological growth phenotype and are smaller in size compared to wild-type controls^22,24^. In addition, Bcl2 is also crucial for the function of osteoblasts, osteocytes and osteoclasts and it has been shown that Bcl2^–/–^ mice demonstrate impaired bone formation and lower turnover of bone metabolism^24^. Other studies have reported that ablation of Bcl2 suppresses osteoclasts’ activity, and decreases proliferation but increases differentiation in osteoblasts, thus resulting in increased bone mass^34,35^. Thus, the complex role of Bcl2 in bone physiology and development emphasizes the need to monitor any potential bone-related side effects of other novel drugs that inhibit BCL-2.

The design of venetoclax as a selective BCL-2 inhibitor followed after the development of other BCL-2 inhibitors (i.e. navitoclax) which were also shown to target BCLX_L_, causing concentration-dependent thrombocytopenia^20^.Venetoclax is currently tested in clinical pediatric cancer studies and according to preliminary results, it showed promising efficacy but was also associated with Grade 3/4 adverse events including anemia, febrile neutropenia, and thrombocytopenia, thus posing a challenge for alternative dosing regimens^36^. However, no clinical data on the potential negative effects of venetoclax on bone tissue are available yet. Notably, the effects of other selective inhibitors that also target developmental pathways have been highlighted in studies investigating the hedgehog pathways inhibitors vismodegib and sonidegib which were shown to cause irreversible growth plate fusion and growth arrest in prepubertal children treated for medulloblastoma^3,37^.

It is important to recognize several limitations of the present study. Firstly, the in vivo experiments were only performed in female mice and we do not know if males would respond similarly. Secondly, our studies were performed in a murine model where growth plates do not fuse in contrast to the human growth plate which fuses in late puberty. Thirdly, we did not address whether there is a potential for catch-up growth after the cessation of venetoclax treatment. Still, this question was not a primary objective of this study, which was designed to investigate any direct effects of venetoclax treatment on bone tissue. Finally, the concentrations of venetoclax used in our in vitro and ex vivo experiments were clinically relevant but were calculated based on concentrations measured in the serum of adult patients, as pharmacokinetic data from pediatric populations remain unavailable^38^.

To our knowledge, this is the first study investigating the effects of a BCL-2 inhibitor on bone growth. Taken together, our study provides evidence that BCL-2 inhibition impairs the function of growth plate chondrocytes leading to impaired longitudinal bone growth and growth plate abnormalities. As venetoclax is being introduced in therapeutic protocols of childhood cancer, either as monotherapy or in combination with other drugs, it is crucial not only to identify the patients that may benefit from such therapies but also to increase our understanding regarding any potential side effects on the growing skeleton that may require intensive monitoring of bone growth. Nevertheless, our findings should be interpreted with caution and more rigorous translational studies including cancer disease models are warranted to elucidate the role of venetoclax on growing bones.

## Supplementary Information

Supplementary Information.

## Data Availability

The data generated in this study are available upon request from the corresponding author.
