# Supplementary material for: Pharmacological inhibition of BCL-2 with the FDA-approved drug venetoclax impairs longitudinal bone growth
Source: Sci Rep. 2023 May 17;13:8054. doi: 10.1038/s41598-023-34965-4 (PMC10192431; doi:10.1038/s41598-023-34965-4)

Pharmacological inhibition of BCL-2 with the FDA-approved drug venetoclax impairs longitudinal bone growth

Lilly Velentza [1] *, Malin Wickström [2], Per Kogner [2,3], Claes Ohlsson [4], Farasat Zaman [1 #], Lars Sävendahl [1, 3 #]

Supplementary information

Compound preparation

Venetoclax (MedChemExpress, Sweden) was diluted in DMSO for the *in vitro* experiments and stock solutions were stored at -20°C. For the *in vivo* experiments, venetoclax was formulated in 60% PHOSAL 50PG, 30% PEG-400, and 10% ethanol and was prepared fresh on the day of treatment.

Supplementary Table 1. List of antibodies used in the study

**Target**	**Target**	**Identifier**	**Host species**
PCNA	Abcam	Cat# ab18197, RRID:AB_444313	rabbit
Bax [E63]	Abcam	Cat# ab32503, RRID:AB_725631	rabbit
ColX	Abcam	Cat# ab58632, RRID:AB_879742	rabbit
Humanin	Novus	Cat# NB100-56877, RRID:AB_838379	rabbit
caspase-3 p20 (N-19)	Santa Cruz	Cat# sc-1226, RRID:AB_630986	rabbit
Bcl-2	Abcam	Cat# ab196495, RRID:AB_2924862	rabbit

Supplementary Table 2. Patients' characteristics

	Age	Sex	Tanner stage	Diagnosis
**Patient 1**	13 yrs	female	4	Constitutional tall stature
**Patient 2**	15 yrs	female	4	Constitutional tall stature
**Patient 3**	12 yrs	male	2	Constitutional tall stature

Supplementary figure 1: Effects of venetoclax treatment on Bax protein expression. Representative immunohistochemical images showing impaired Bax expression in the fetal rat metatarsals treated with venetoclax or DMSO (control). Alcian blue was used as counterstain. Magnification 20x, scale bar= 100 µm.

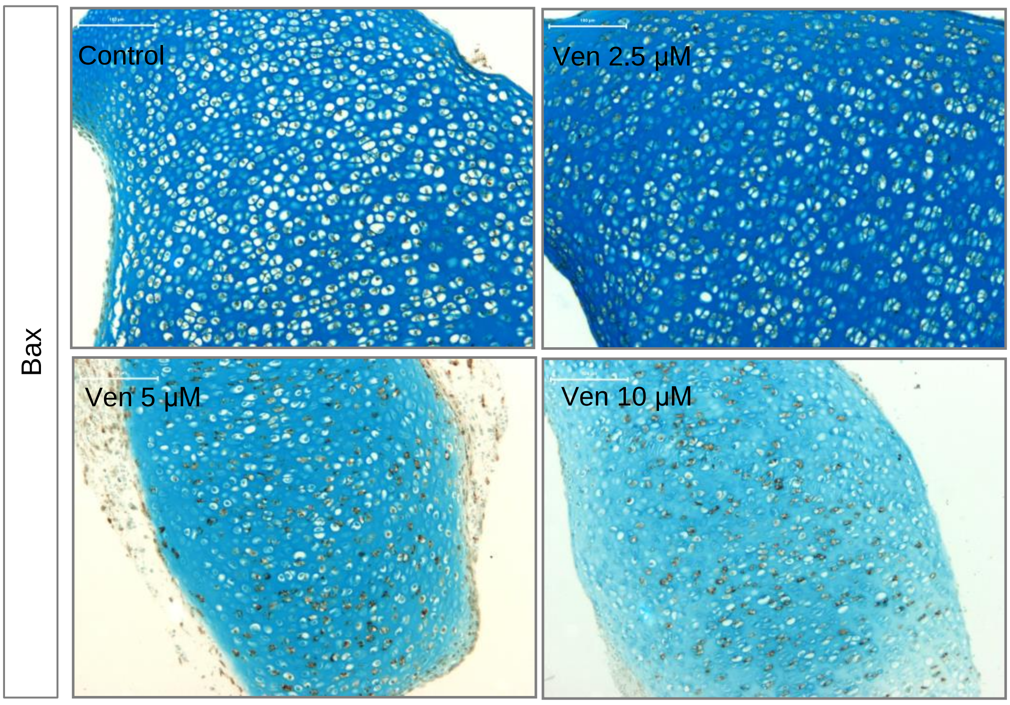

Supplementary Figure 2: Effects of venetoclax treatment on PCNA protein expression in tibial growth plates. Representative immunohistochemical images and quantification of PCNA protein expression in control and venetoclax-treated mice. Alcian blue was used as counterstain. Magnification 40x, scale bar= 50 µm. Mean values ± SD are shown (n=4-5/group).

Suppl. Fig.2

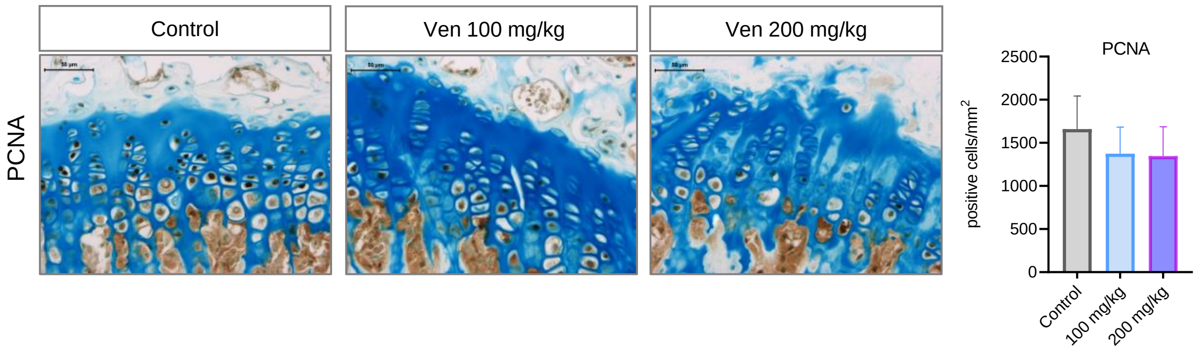

Supplementary Figure 3: Effects of venetoclax treatment on Bax protein expression in tibial growth plates. Representative immunohistochemical images and quantification of Bax protein expression in control and venetoclax-treated mice. Alcian blue was used as counterstain. Magnification 40x, scale bar= 50 µm. Mean values ± SD are shown (n=4-5/group).

Suppl. Fig.3

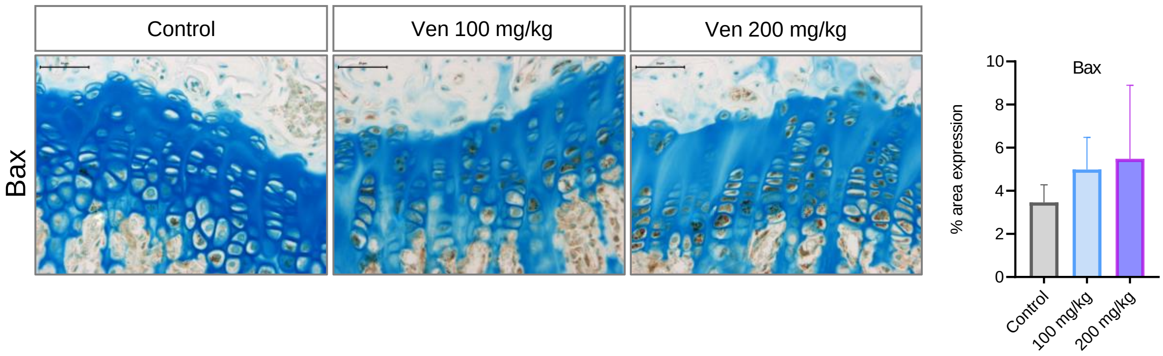

Supplementary Figure 4: Effects of venetoclax treatment on ColX protein expression in tibial growth plates. Representative immunohistochemical images and quantification of ColX protein expression in control and venetoclax-treated mice. Alcian blue was used as counterstain. Magnification 40x, scale bar= 50 µm. Mean values ± SD are shown (n=4-5/group).

Suppl. Fig.4

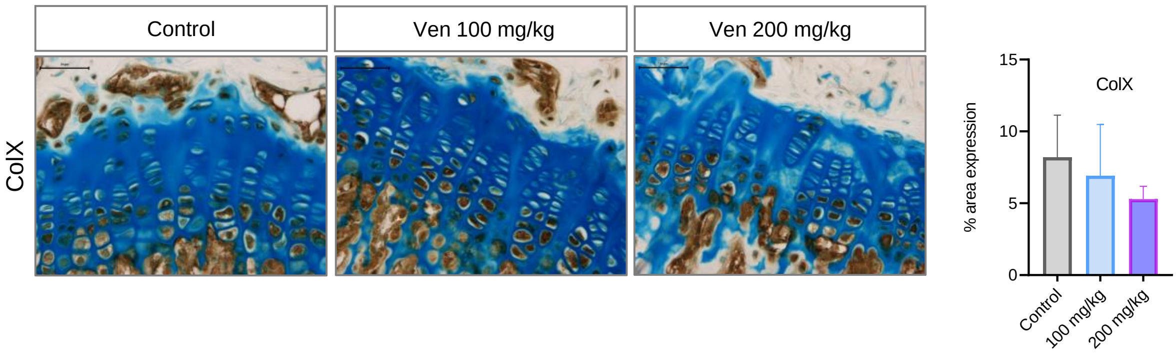

Supplementary Figure 5: Effects of venetoclax treatment on caspase-3 protein expression in femoral growth plates. Representative immunohistochemical images and quantification of caspase-3 protein expression in control and venetoclax-treated mice. Alcian blue was used as counterstain. Magnification 20x, scale bar= 100 μm. Mean values ± SD are shown (n=4-5/group).

Suppl. Fig.5

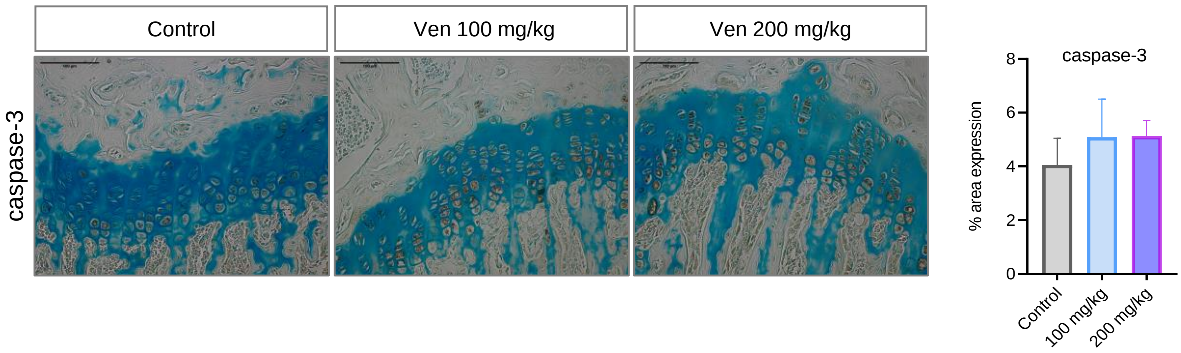

Supplementary figure 6: Representative immunohistochemical images showing the baseline BCL-2 expression in the fetal rat metatarsals and tibial growth plates. Alcian blue was used as counterstain. Magnification 20x (tibia) and 10x (metatarsal), scale bar= 100 μm.

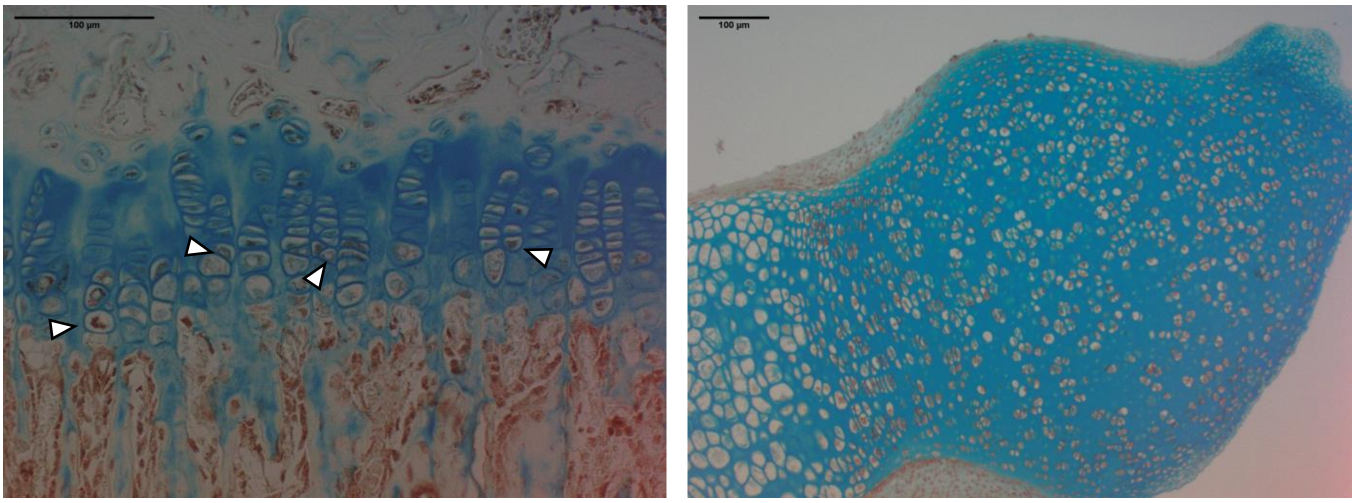

Supplement: Supplementary file 1 — Supplementary Information. [file 41598_2023_34965_MOESM1_ESM.pdf]
